# New Polyphenol-Containing LDL Nano-Preparations in Oxidative Stress and DNA Damage: A Potential Route for Cell-Targeted PP Delivery

**DOI:** 10.3390/ma13225106

**Published:** 2020-11-12

**Authors:** Hanna Lewandowska, Monika Kalinowska

**Affiliations:** 1 Centre for Radiation Research and Technology, Institute of Nuclear Chemistry and Technology, 16 Dorodna St., 03195 Warsaw, Poland; 2 Department of Chemistry, Biology and Biotechnology, Faculty of Civil Engineering and Environmental Sciences, Bialystok University of Technology, Wiejska 45E Street, 15351 Bialystok, Poland; m.kalinowska@pb.edu.pl

**Keywords:** polyphenols, low-density lipoprotein, nanoparticles, drug delivery, oxidative stress

## Abstract

Low-density lipoprotein (LDL) preparations of the chosen polyphenols (PPs) were prepared for the first time in the literature. The solubility of the PPs in the lipidic core of the LDL increased with the increase of their lipophilicity. The anti-/pro-oxidative properties and toxicity of LDL-entrapped PPs toward A 2780 human ovarian cancer cells were examined. The obtained preparations were found to be stable in PBS, and characterized by low toxicity. A binding affinity study revealed that the uptake of PP-loaded LDL particles is non-receptor-specific under experimental conditions. The antioxidative potential of the obtained PPs-doped LDL preparations was shown to be higher than for the PPs themselves, probably due to facilitating transport of LDL preparations into the cellular milieu, where they can interact with the cellular systems and change the redox status of the cell. The PPs-loaded LDL displayed the highest protective effect against Fenton-type reaction induced oxidative DNA damage.

## 1. Introduction

Polyphenols, defined as compounds exclusively derived from the shikimate/phenylpropanoid and/or the polyketide pathways, featuring more than one phenolic unit, and deprived of nitrogen-based functions, comprise a very broad group of chemicals, and are widely distributed in plant foods [1]. In animals, polyphenols (PPs) are metabolized following ingestion in the stomach, small and large intestine, and liver, and therefore are poorly available in cells in their native form [2,3]. Despite this, their regulatory and pro-oxidative action has been widely studied, and it is currently believed that PPs have beneficial antioxidant effects at low, and pro-oxidative and cytostatic effect at higher, doses [4,5,6]. PPs protect against carcinogenesis by promoting apoptosis, decreasing cellular proliferation, and inhibiting inflammation, thus hampering angiogenesis and metastasis (reviewed in [7]). PPs protect against DNA damage and tumor initiation by modulating phase I and II xenobiotic-metabolizing enzymes, and influence epigenetic modifications and the dynamic state of chromatin [7,8,9,10]. On the other hand, as stated in [5], high doses of polyphenols may damage DNA. Similarly, according to our earlier results [11], in plasmid DNA cleavage electrophoretic tests, quercetin promoted DNA damage (single- and double-strand breaks), at 5 µM and 50 µM concentrations. Curcumin also displayed this pro-oxidative effect, but only in a higher concentration (of 50 µM). For the lower, 5 µM concentration, an altogether lower antioxidative effect could be seen, with a considerable reduction of double-strand brakes (decreased L-form of plasmid), and a negligible increase of the single strand brakes (OC form) compared to the control sample. Chlorogenic acid proved a potent antioxidant against DNA ROS-induced damage in both high and low concentrations. In conclusion, PPs display a spectrum of mild anti- to pro-oxidative potential, and the final effect also depends on their concentration.

In view of the many regulatory functions PPs were shown to exhibit in mammalian cells and their potential beneficial effect to human health, attempts are ongoing to improve the water solubility and bioavailability of PPs. In the study of Nguyen [12], curcuminoid glycosides of stevioside (Ste), rebaudioside A (RebA), or steviol glucosides (SG) were solubilized in water. The optimum extraction condition by Ste, RebA, or SG resulted in 11.3, 9.7, or 6.7 mg/mL water-soluble curcuminoids. Apart from chemical modifications of the PPs many attempts have focused on looking for the appropriate drug carriers. Among the successful solutions are the liposome [13], polymeric micelles [14], phospholipids [15], and other nanoparticle-based drug delivery systems [16]. For instance, for one such nanoformulation, oral administration was demonstrated to yield more than 30-fold higher bioavailability compared with conventional curcumin in rat models [17]. In healthy human subjects, the maximum serum curcuminoid concentration after administration of 30 mg of water-soluble curcuminoids was 30 ng/mL, compared to 2 ng/mL after conventional curcumin administration [17].

As an alternative to the above-mentioned synthetic and semi-synthetic vehicles for PPs delivery, we proposed a fully natural carrier capable of carrying the lipophilic payload. Low-density lipoprotein (LDL) has attracted tremendous attention for its applications in cargo loading and delivery, owing to its unique features: LDL, as the major ligand for the low-density lipoprotein receptors (LDLR) that are highly expressed in quickly proliferating cells, allows moderate uptake of the drug in normally proliferating cells, and a highly increased uptake in cancer, a feature that potentially makes it an ideal carrier for PPs, which were shown in the literature to have beneficial antioxidant effects in low, and pro-oxidative and cytostatic effects in higher doses [4,5,6]. Another advantage connected to the application of LDL as a drug carrier is it’s large (ca. 20 nm diameter) size. While normal tissue is not permeable to big particles, tumor tissue tends to accumulate macromolecules and especially lipids. This phenomenon has been characterized and termed the tumor-selective enhanced permeability and retention (EPR) effect [18]. The enhancement of vascular permeability plays a critically important role in tumor growth, in facilitating an adequate supply of nutrients, and possibly oxygen, to meet the great demands of the rapidly growing tumors. Therefore the EPR concept is now regarded as a “gold standard” in the design of new anti-cancer agents. The next attractive feature of LDL as a bio carrier is its biosafety. While synthetic materials can cause toxicology problems, the lipoproteins are fully biodegradable in the body by way of natural mechanisms. Moreover, LDL particles are solid particles. Consequently, they have greater structural stability than, for example, the liposomes that are currently a very popular medium for PPs in medical preparations [13]. Taking into account the above-described findings and conclusions, it seems a reasonable scientific goal to look into the potential of LDL as carriers of polyphenolics, and into the effects that PP-loaded LDL preparations have in cells.

In this work, new polyphenol-containing LDL nano-preparations were proposed. Chlorogenic acid (CLA), quercetin (Q), and curcumin (CUR) were chosen because these PPs attract much attention from the pharmaceutical industry and supplement market as potential anti-cancer compounds, antioxidants, enzyme regulators, and nutraceuticals [19,20]. Nevertheless, the poor bioavailability of Q and CUR hampers its action in vivo [21]. Moreover, these three representatives of PPs, which are widely present in popular and scientific consciousness, constitute a logical series, due to the linearly increasing value of logP from CLA to CUR (Table 1). Lipophilicity, to a large extent, determines the effectiveness of a pharmaceutical [22,23]. Modulation of lipophilicity through the use of carriers allowed for excellent improvement of the therapeutic properties of such drugs as paclitaxel and doxorubicin (Abraxane [24], Doxil [25]). The proposed logical series of PPs, superposed with the fully natural lipophilic carrier, present an attempt to find optimal conditions for administering drugs with different lipophilicities, based on the specific example of popular dietary supplements. In this work, the procedure of synthesis of PP-saturated LDL nanoparticles was described, and their anti-/pro-oxidant activity and toxicity toward A 2780 human ovarian cancer cells were tested in neutral red uptake (NRU), dihydrorhodamine (DHR) oxidation, DiL staining of lipoproteins, LDL binding, and plasmid nicking assays.

## 2. Materials and Methods 

### 2.1. Materials 

All reagents were purchased from Sigma Aldrich, unless otherwise indicated. Human plasma used for the tests was collected by the Regional Center for Blood Donation and Blood Treatment (Warsaw), operating in accordance with the Act of August 22, 1997 on the public blood service (Polish legal act in accordance with the Declaration of Helsinki).

### 2.2. Isolation and Purification of LDL

LDL was isolated by sequential ultracentrifugation in the KBr gradient from the fresh human plasma obtained from healthy volunteers, after the reduction of pathogens according to the modified method of Havel et al. [26]. LDL fraction was desalted on GE-PD-10 (GE Healthcare, 17–0851-01, Chicago, IL, USA) columns by washing with PBS and sterilized by filtration with 0.22 mm filters. The preparations were sealed under nitrogen and stored in the dark at 4 °C. The storage time never exceeded 2 weeks. The LDL particle size and dispersity were determined by dynamic light scattering (DLS), whereas protein contents were determined according to the modified method of Lowry [27]. The LDL concentration is expressed in protein content per mL throughout the article.

### 2.3. DLS Particle Size Measurement 

To measure the size of the native and modified LDL particles, the diffusion barrier method (DBM) was used. A folded capillary cell was filled with 700 µL of PBS. Then using a gel electrophoresis loading tip, a small aliquot of the sample (typically 100 µL, ca., 6 mg/mL of protein, as measured by Lowry) was introduced into the bottom of the cell, being careful not to introduce any air bubbles. The size was measured with a Zetasizer Nano ZS (Malvern, PA, USA). The measurement was set to the backscattering mode. The initial setup of the experiment was adjusted with regard to sample turbidity so that the attenuation reached values within the range of 5–8. 

Development and optimization of the method of saturating LDL with polyphenols (PPs) quercetin (Q), curcumin (Cur), chlorogenic acid (CLA): Saturated solutions of Q, Cur, and CLA in water and methanol were obtained, maxima of UV/VIS absorption were determined, and molar extinction coefficients in methanol were determined. To achieve this, the PPs were added in excess to the solvent in anaerobic conditions and kept in the closed vials under stirring for 24 h at 35 °C. Next, the temperature was lowered to RT, the mixture was passed through Whatman® filter paper, and the solution was measured spectrophotometrically. An aliquot of the solution was then dried under vacuum, and the masses were weighed (data not shown). On that basis, the concentrations suitable for the extinction coefficients were chosen. To measure the molar extinction coefficients, Q and CLA were dissolved to a concentration of 25 µM, and Cur to 12,5 µM, and the extinction coefficients were calculated from the absorbance at 372 nm for Q, 422 nm for Cur, and 329 nm for CLA. The LDL saturation method with curcumin, quercetin, and chlorogenic acid was optimized. For this purpose, the obtained LDL preparations were incubated with the above-mentioned polyphenols for 2 h and 11 h, at various concentrations of PPs (finally selected 0.5 mg PP/mL LDL at the LDL conc. 6–9 mg/mL. It should be noted, that the solubility of Q and Cur is higher in culture medium than in water [28]) under various temperature conditions, with or without stirring (finally room temperature was selected, with mixing on a shaker, in the dark, 11 h). Samples were purified on Sephadex G-25 PD-10 columns (GE Healthcare, Chicago, IL, USA). PPs content was determined in purified preparations. The preparations were extracted with methanol (1:9 *v/v* ratio of LDL to MeOH), and the obtained extract was determined for polyphenol content by the UV/VIS method, based on the obtained extinction coefficients. Native and polyphenol-saturated nanoparticle size measurements were made using the DLS method, to evaluate the effect of polyphenols on LDL size.

### 2.4. Cell Culture Conditions

Human ovarian cancer A 2780 cells were purchased from ATCC (Manassas, VA, USA) and cultured in RPMI medium containing 10% FBS and Pen-Strep, and grown under standard conditions [29]. For the NRU [30] and DHR [31] tests, cells in the logarithmic phase of growth were seeded in 96-well culture plates and cultured at 37 °C under a 5% CO_2_ atmosphere for 24 h. The initial number of cells per well was 8000 for all tested samples. The cultured medium was removed when the cells adhered to the plate wall. The cells were then incubated in 100 µL of medium with the growing concentrations of the studied compound for 48 h. Non-treated cells were used as the control.

### 2.5. Neutral Red Uptake (NRU) Assay of Cell Metabolic Activity 

Curcumin and quercetin concentrated DMSO solutions were diluted in the culture medium to obtain preparations containing 70 µM of PPs. Cells were grown in a 96-well plate (TPP, Switzerland) to the density of 1 × 10^4^ cells/well, the Q, Cur, QLDL, CurLDL, or LDL solutions were added to the culture medium. Namely, the preparations were diluted to obtain a series of two-fold dilutions in the range 70–0.27 µM of PPs. Then, 50 µL of these solutions were added to the cell-culture (96-well plates, 100 µL of cell culture volume). For the pure LDL preparation, the concentration was in the range 1–0.004 mg/mL LDL, which corresponded to the LDL concentration of the other preparations: the 23 µM PP concentration corresponded to 1 mg/mL and 0.785 µg/mL LDL concentration for Q and Cur, respectively. The cells were incubated with the drug for 24 h. Then, the drug-containing medium was removed, the cells were washed with PBS, and incubated for 3 h with 100 µL/well of neutral red (NR) solution (3 mg/mL) at 37 °C. At this time, the dye solution was removed, and the cells were lysed in 1% glacial acetic acid, 50% ethanol, and 49% H_2_O solution. Fluorescence was measured at Ex/Em 530/645 nm, respectively, in a plate reader spectrophotometer Infinite M200 (Tecan, Grödig, Austria). 

### 2.6. Determination of the Level of Reactive Oxygen Species in a 2780 Human Ovarian Cancer Cells 

A Dihydrorhodamine (DHR) oxidation test was performed according to [32]. Reactive oxygen species (ROS) level was measured in A2780 cells incubated with sole PPs, or their LDL preparations, after 48 h incubation, and related to the ROS level in the control cells. Briefly, cells were treated with 23 µM of PPs, PP-saturated LDL nanoparticles (PPLDL), or 0.5 mg/mL pure LDL, in 96-well plates for 48 h. After co-incubation with the studied preparations, the cells were washed twice with PBS, and the freshly prepared DHR working solution (10 µM) in serum- and phenol red-free medium was added. The cells were incubated for 20 and 40 min. After incubation, the level of reactive oxygen species (ROS) was measured fluorimetrically on a Tecan multi-plate reader (Tecan, Männedorf, Switzerland) at Ex/Em wavelengths of 485 and 528 nm, respectively. The LDL preparation of 0.5 mg/mL was used as a control.

### 2.7. DiL Staining of Lipoproteins 

In order to evaluate the uptake of native or modified lipoproteins by A2780 cells, the LDL preparations were stained with a lipophilic fluorescent dye, 1,1’-dioctadecyl-3,3,3’,3-tetramethylindocarbocyanine perchlorate (DiL stain, Molecular Probes D282) [33]. The LDL preparations (6–8 g/L protein) were anaerobically treated with 3 g/L Dil in DMSO to a final DiL conc. 75 mg/L. The preparations were incubated for 18 h at 35 °C, in the dark. Subsequently, LDL was passed through a GE PD-10 column in anaerobic conditions to remove the redundant DiL. To achieve anaerobic conditions, the columns and the buffer were deaerated in a glovebox MBraun Labstar (MBraun, Garching, Germany), O_2_ level <1 ppm for 24 h and the LDL solution was evacuated three times in the antechamber. The protein content and the fluorescence at 520/580 nm per mg of protein were determined.

### 2.8. LDL Binding Assay 

A2780 cells at the density of 10^4^ were seeded in the 96-well plates and cultured at 37 °C under 5% CO_2_ atmosphere for 24 h. Then, the cells were incubated with the increasing amounts of DiL-stained QLDL, CurLDL, or LDL for 2 h at 4 °C in a standard medium. The amounts of the added lipoproteins were in the range of 1–580 nm. Next, the medium was removed, and the cells were washed twice with PBS. Cells were then lysed in DMSO (150 µL per well) for 20 min with agitation, and the fluorescence was measured at 520/580 nm in a plate reader spectrophotometer Infinite M200 (Tecan, Grödig, Austria) [34,35].

### 2.9. Plasmid Nicking Assay 

To estimate how the polyphenols (PPs) influence the reactive oxygen species (ROS)-mediated DNA damage, a plasmid cleavage test was carried out. The assay was based on the measurement of the abundance of DNA bands corresponding to the supercoiled (CCC), open circular (OC), and linear (L) forms of the plasmid visualized after electrophoresis, that is directly related to the extent of DNA damage [36,37]. Plasmid pUC19 was incubated at 22 °C for 10 min in dark, anaerobically, in the presence of 700 µM H_2_O_2_ and 0.7 µM Fe^2+^ ions (in order to induce Fenton-type reaction mediated ROS damage of DNA). The H_2_O_2_ and Fe ions containing samples were also incubated in the presence of quercetin (Q), quercetin-loaded LDL (QLDL), curcumin (CUR), curcumin-loaded LDL (CURLDL), or the native LDL (0.55 mg/mL). The LDL concentrations in the PP-loaded LDL were 0.21 mg/mL for QLDL and 0.88 mg/mL for CurLDL. The final concentration of the PPs in all preparations was 5 µM. Native pUC19 plasmid and pUC19 cleaved L form were used as a negative and positive control, respectively. The incubation mixture contained 44 ng pUC19 plasmid DNA. The reaction products were resolved electrophoretically on 1.5% agarose gel, containing 0.25 μg/mL ethidium bromide. To obtain a linear form, the plasmid was cleaved with SmaI endonuclease (Fermentas). The DNA bands were visualized under UV light, photographed, and the bands’ intensity was estimated by ImageJ software (http://rsbweb.nih.gov/ij/index.html). The optical density was measured for each line, and the percentage share of CCC and OC bands in the total optical density for a given sample was evaluated.

## 3. Results

**Figure 1:**
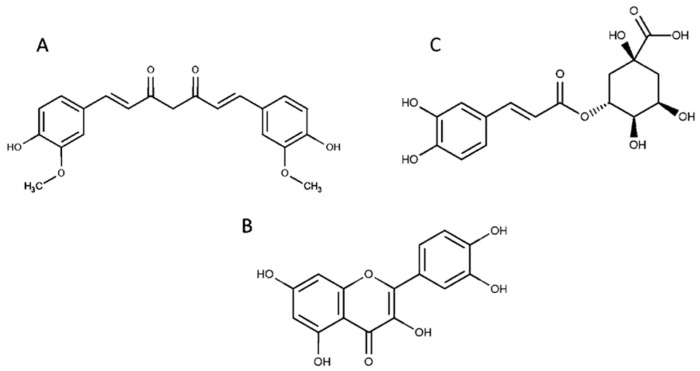
Structure of curcumin (**A**), quercetin (**B**), and chlorogenic acid (**C**).

The three PPs, quercetin (Q), curcumin (Cur), and chlorogenic acid (CLA) (Figure 1), varying with solubility were chosen for the tests. 

**Figure 2:**
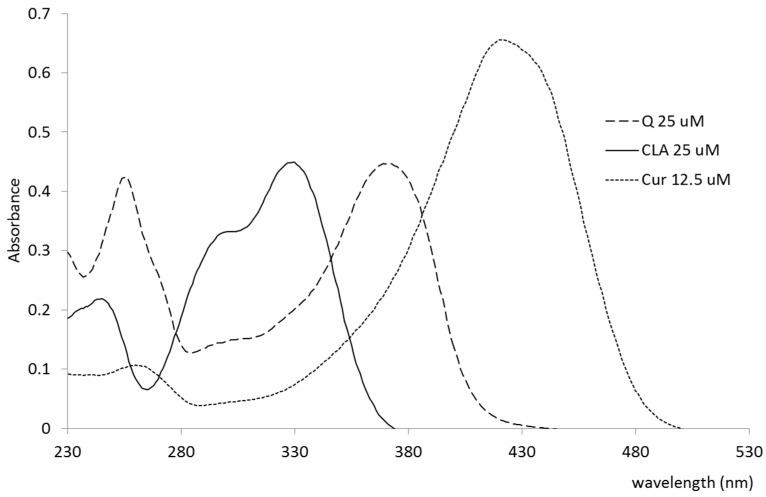
UV/VIS spectra of quercetin (Q), curcumin (Cur), and chlorogenic acid (CLA) in methanol.

The polyphenols were dissolved in methanol in order to obtain their absorption maxima and the molar extinction coefficients for further measurements. The UV/VIS spectra of the polyphenols (PPs) in methanol in the range of 230–600 nm are shown in Figure 2. Table 2 summarizes molar extinction coefficients at the respective maximum absorption wavelengths in methanol.

### 3.1. Influence of PP Saturation on the Size of the LDL Particle

**Figure 3:**
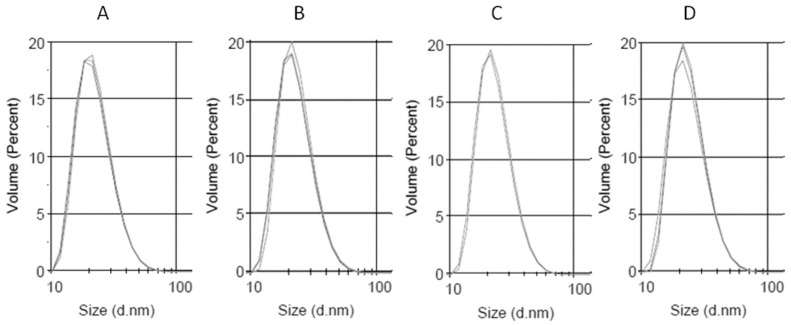
Dynamic light scattering (DLS) measurement of the size distribution of the obtained low-density lipoprotein (LDL) preparations. (**A**) LDL, (**B**) QLDL, (**C**) CurLDL, and (**D**) CLALDL. PPs-doped LDL samples were prepared by mixing 0.5 mg of PPs per 1 mL of native LDL preparation (6 mg/mL of protein), followed by 11 h incubation at RT, with shaking in the dark. Three repetitions of measurements are overlaid for each preparation. The full diagrams in the range 0.1–10,000 nm have been included in Supplementary Material, Supplementary Figures S1–S4.

The size of the obtained preparations was measured using DLS, and the PP-loaded LDL preparations were compared to the native LDL. The obtained LDL preparation characterized with a good monodispersity, having the polydispersity index of 0.159. The main diameter by volume was 22.8 nm. The DLS measurement data for the obtained LDL preparation are given in Figure 3, Table 3. These preparations were further used for saturation with Cur, Q, and CLA. It turned out, that the LDL was best saturated with the PPs, when the LDL solution was gently agitated over the PPs solid powder (0.5 mg per mL of LDL, 6 mg/mL of protein), anaerobically at room temperature. After being saturated with PPs, the LDL particles displayed similar diameters, as untreated LDL, as shown in Table 2 and Figure 3. 

**Figure 4:**
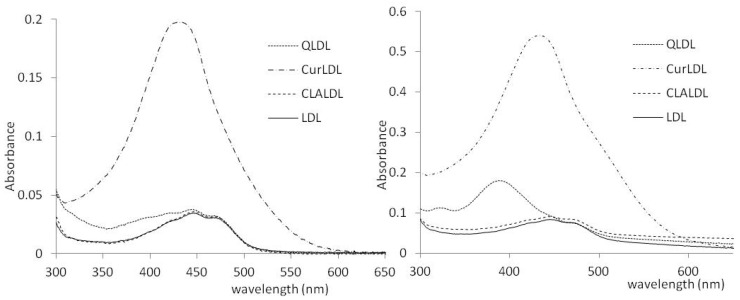
The UV/VIS spectra of the PPs obtained from the LDL preparation methanol extracts. Left: after 2h, Right: after 11 h.

The obtained preparations were tested for polyphenol content. The methanol extracts of PPLDL preparations gave the clearly visible spectra of Q and Cur, extracted from the LDL core. CLA was not found in the CLA-treated LDL, either after 2 or 11 h of incubation. The spectra of the methanol-extracted PPs from LDL preparations (11 h incubation) are given in Figure 4. The PPs contents found in the LDL preparations, as calculated from the found ε values after subtraction of native LDL extract spectrum, are listed in Table 4.

### 3.2. Toxicity

**Figure 5:**
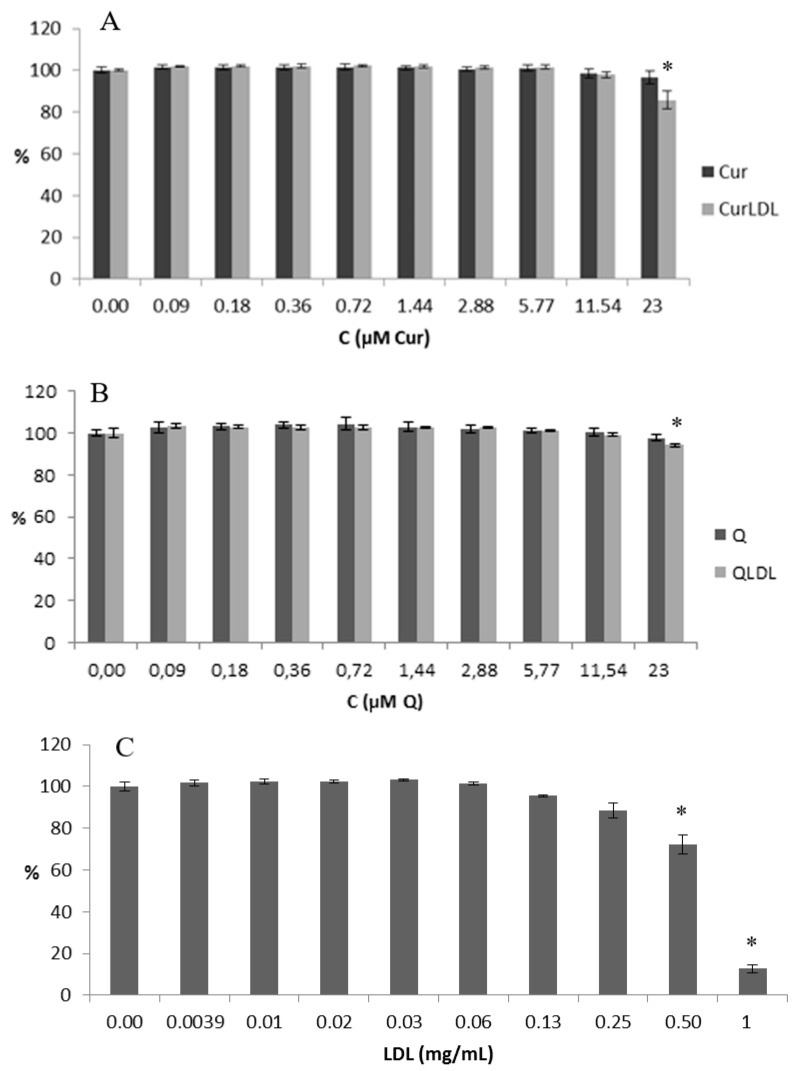
The metabolic activity in cells treated with increasing concentrations of PP preparations and the native LDL preparation. (**A**,**B**) Concentrations of PPs varied from 23 to 0.09 µM, (**C**) the concentration of LDL varied from 1 to 0.004 mg/mL (by protein concentration, i.e. 1.8 µM to 7 nM of ApoB100), which corresponds to LDL concentration in PP-saturated LDL samples. The statistically significant decrease is noted by an asterisk (*p* < 0.05).

Due to the fact, that CLA was not found in the respective LDL preparations, only QLDL and CurLDL were further examined for their anti-/pro-oxidative properties and toxicity. In that regard, a toxicity assay was performed. The NRU metabolic activity test was performed, as described in the materials and methods, for the decreasing concentrations of PPs, as indicated in Figure 5.

The NRU test is an indirect test of cell viability. It is based on the ability of cells to uptake the neutral red (3-amino-7-dimethylamino-2-methylphenazine hydrochloride, CAS 553-24-2) dye by active transport, where live cells incorporate neutral red into their lysosomes. As cells begin to die, their ability to incorporate neutral red diminishes. Thus, the loss of neutral red uptake corresponds to the loss of cell viability [38].

The native LDL decreased the cellular proliferation to a higher degree than all the polyphenol-containing preparations, and in a statistically significant level in all cases. QLDL decreased the proliferation of the cells to a higher extent than the pure Q, and the difference was statistically important (*p* < 0.05). CurLDL decreased the proliferation of the cells to a higher extent than the pure Cur, but this difference was not statistically important. 

### 3.3. LDL Binding Affinity Test

**Figure 6:**
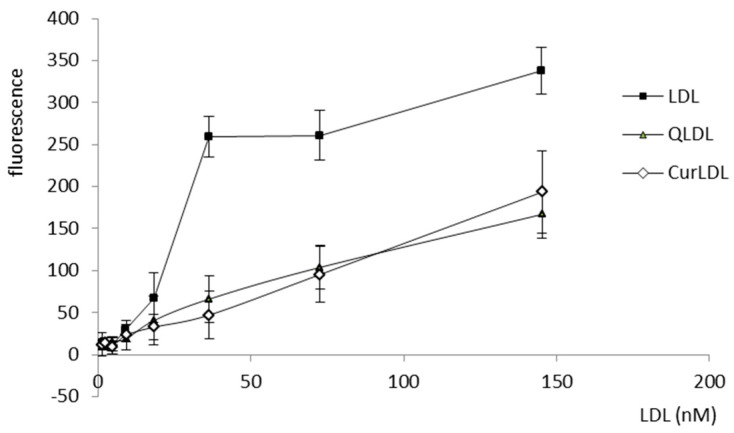
The A2780 positive cells were exposed to increasing concentrations of DiL-stained LDL preparations, as indicated. Error bars represent SD values.

LDL was bound by the A2780 cells, with a logarithmic response to the increasing concentrations of LDL. Otherwise, the QLDL and CurLDL binding did not exhibit a logarithmic character, showing instead a linear binding dependence on protein concentration (Figure 6).

### 3.4. The Level of the Reactive Oxygen Species

**Figure 7:**
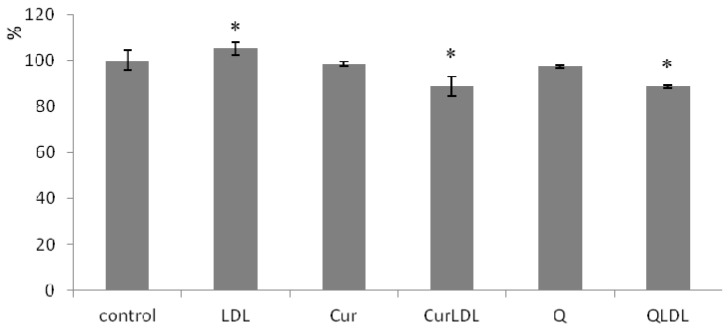
The level of reactive oxygen species (ROS) in A2780 cells incubated with 23 µM preparations of PPs. Cells were treated with PP preparations for 48h, ROS level measured after 20 min from DHR addition. Error bars represent SD values. The asterisks denote statistically important differences *versus* control at the significance level 0.05 (T.test, p-values: LDL 0.026; CurLDL 0.001; QLDL 0.030).

The level of ROS is presented in Figure 7. It can be seen, that LDL treatment increased the level of ROS in a statistically significant manner. Contrariwise, the treatment with LDL-entrapped PPs decreased the ROS level, not only to the base level for untreated cells, but caused a decrease in ROS levels significantly below the level found in the untreated control. At the same time, pure PPs did not affect ROS levels to a statistically significant degree.

### 3.5. Plasmid Nicking Assay

**Figure 8:**
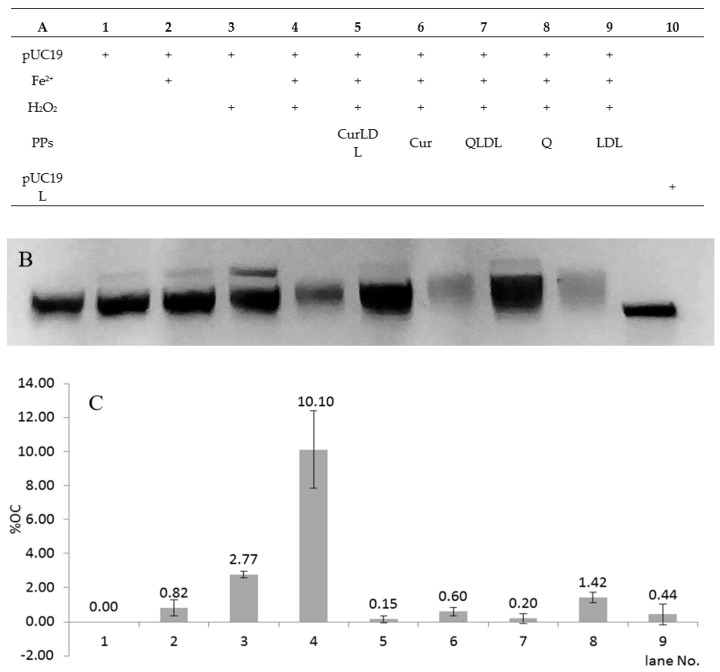
ROS-induced DNA damage caused by the Fe^2+^/H_2_O_2_ redox system in the presence or absence of polyphenols. (**A**): the treatment applied for each sample; (**B**): representative gel image; (**C**): values for the OC band intensities in the graph; the linear form of pUC19 is shown as a reference in line 11 (B). Concentrations: FeSO_4_ 0.7 µM, Q 5 µM, Cur 5 µM, LDL line 5 0.18 g/L, lines 7–9 0.21 g/L. H_2_O_2_ 700 µM, where applicable, pUC19 44 ng/µL per sample. Error bars represent SD values, data labels represent % of OC band abundance. *P*-values versus LDL: Q 0.026*; CUR 0.713; QLDL 0.403; CURLDL 0.340; *p*-value Q/QLDL 0.040*; p-value CUR/CURLDL 0.119; LDL versus positive control *p*-value 0.009*.

Figure 8 presents the chart for the sample treatment, the resolved gel, and the percentage of each form of the plasmid found in the samples after treatment. As expected, the treatment of pUC19 with 700 µM H_2_O_2_ in the presence of 0.7 µM Fe^2+^ ions, in comparison to the damage induced by Fe^2+^ and H_2_O_2_, resulted in the emergence of an additional plasmid band, migrating slower under the electric current in the agarose gel (Figure 8). This band was assigned to the OC form of the plasmid [36]. The band was calculated by optical density measurement of the gel image to represent a 10% abundance of the total observed plasmid (±2.3% between experiments). After treatment with H_2_O_2_ and Fe^2+^ in the presence of 5 µM PPs, in all the PPs-treated samples a pronounced lowering of OC form was observed. The OC form abundance was reduced back to below 1.5% in all samples containing PPs. The OC form was also reduced in the sample containing the native form of LDL. The OC form reduction was statistically significant between the samples treated with PPs or LDL, versus the non-treated sample in the presence of H_2_O_2_ and Fe^2+^. The differences in OC abundance were not significant between the PPs or LDL-treated samples. No double-strand breaks were observed under experimental conditions. The single-stranded form of the plasmid is present in line 10 as a reference. Interestingly, in the samples containing LDL, some amount of the plasmid was seen as a fluorescence close to the starting point on the agarose gel (not shown). This effect was not observed for the samples not containing LDL. This might have been due to the interaction of the plasmid with LDL particles. Therefore, the LDL-containing preparations showed lower fluorescence due to the lower amount of migrating DNA present in bands after electrophoresis.

## 4. Discussion

Lipophilicity is a major determining factor in a compound’s absorption, distribution in the body, penetration across vital membranes and biological barriers, metabolism, and excretion. Depending on the administration route of a given compound and its target milieu in the biological environment, an ideal candidate for a drug must have lipophilicity allowing for penetration through the relevant barriers [22,23]. Lipophilicity also affects formulation, dosing, drug clearance, and toxicity. Though it is not the only determining factor in these issues, it plays a critical role in helping scientists limit the liabilities of new drug candidates. Lipophilicity determines the route of drug administration. The natural PPs, quercetin (Q), curcumin (Cur), and chlorogenic acid (CLA) tested in this work fall among the most frequently studied polyphenolic compounds. These compounds vary with solubility to a high extent, which is illustrated by their LogP (lipophilicity) values (Table 1).

Herein, the LDL was shown to absorb polyphenols to its lipophilic core. The solubility of PPs in the lipidic core of LDL increased with the increase of their lipophilicity. Curcumin, the most lipophilic of the tested PPs, was found to accumulate in LDL to the highest concentration. The water-soluble chlorogenic acid (approximately 25 mg/mL in H_2_O) was not found in the LDL particle, even after 11 h of coincubation (Figure 4, Table 4). The PPs-doped LDL particles did not change their size compared to the native LDL particle. The lack of aggregation and high monodispersity of PPLDL preparations reveals their high stability (Figure 3, Table 3). 

LDL-entrapped PPs decreased LDL cytotoxicity. All the applied PPs preparations, both pure and LDL-entrapped Q and Cur had a low impact on the metabolic activity of A2780 cells (Figure 5). They were shown to be less toxic than the LDL itself towards A2780 cells in the applied concentration range. The small decrease in proliferation of cells caused by PPs with respect to their LDL-entrapped counterparts reveals, that both Q and Cur showed a lesser impact on cell proliferation (were less cytostatic) than their LDL-entrapped counterparts. Yet, this difference was found statistically significant only for Q. Moreover, Q and Cur dramatically decreased the toxicity of the LDL in the applied experimental conditions. The pronounced difference in the toxicity of the native LDL in comparison to its PPs-doped preparations can be explained in view of the affinity binding study results. They showed, that both the Q- and Cur-enriched LDLs were not bound by the cells in a receptor-dependent manner, as follows from the linear and not logistic dependence of binding versus the protein concentration. Thus, Q and Cur interaction with the LDL ApoB100 protein seems to destroy the receptor binding affinity of LDL, in effect reducing LDL uptake in the cells, followed by the decrease in toxicity. In other words, due to the interaction of PPs with the LDL protein, the active centre of ApoB100 was disturbed or obstructed by the PPs molecules, and thus did not interact properly with the LDL receptor(s). Therefore LDL was taken up by the cells to a lower degree and was toxic in higher concentrations. It has to be noted, that for all the LDL preparations, a considerable receptor non-specific binding was observed under the experimental conditions. In addition, the fact of LDL toxicity is in line with the literature findings. Native LDL shows some toxicity, as can be seen, e.g., in our previous works [34,39]. The LDL toxicity varied between the cell lines. 

The observed reduction of the antiproliferative effect by LDL-entrapped PPs, compared to the native LDL, should be related to the antioxidative effect that was exhibited in the A2780 cells incubated with sole PPs, and their LDL preparations. Even though LDL itself caused a statistically important increase in the ROS levels (in line with its toxicity), the level of the reactive oxygen species (ROS) was significantly lower for the LDL-entrapped PPs, while the sole PPs did not affect ROS levels in a statistically significant manner. The fact that LDL-entrapped Q and Cur provided better antioxidative action in the cells, suggests that the LDL entrapment enhances their transport into the cellular milieu, when they can interact with the cellular systems and organelles, thereby influencing the redox status of the cell. For context, the most lipophilic of the studied PPs, Cur, when administered in its pure form to the cells was shown to be trapped inside the cell membrane resulting in higher local curcumin concentration in membranes than in plasma [40]. Of course, as shown by the uptake test, the LDL-entrapped polyphenols hampered LDL uptake, therefore, reducing its cytotoxicity. Yet, it can be seen that the remaining PPLDL uptake was sufficient to cause a significant ROS level reduction, in comparison to the untreated control. It has to be borne in mind, that the ROS decrease was only 15%, while the toxicity decrease was much more pronounced. As mentioned, the LDL that is not loaded with the PPs binds to the cells with much higher affinity, and the plot from Figure 6 suggests that this binding has a receptor-dependent character (the logistic-like character of the binding plot). Altogether, the ROS effect cannot have been entirely responsible for the diminished toxicity of the LDL. The overall effect of polyphenols in regard to toxicity comes not only from ROS level reduction (which was also shown) but as well is simply an effect of lower LDL uptake. Taken together it appears that the LDL entrapment of Q and Cur enhances their cellular uptake and pronounces antioxidative action of these polyphenols in the tissue culture, and at the same time, it reduces LDL cellular uptake, making it nonspecific and not receptor-dependent. This finding is well in concert with the literature findings, that Cur [41] and Q, as well as other flavonoids [42], interact with numerous proteins in the biological milieu through covalent, non-covalent hydrophobic, and hydrogen bonding interactions, thus modulating their biological activity. 

The antioxidative potential of the obtained preparations was also observed in the reduction of the oxidative DNA cleavage in the plasmid nicking assay. Under the applied experimental conditions, Haber–Weiss-type reactions, induced by the treatment of plasmid with H_2_O_2_ and Fe^2+^, caused single-strand breaks of the pUC19 plasmid, resulting in the 10% abundance of the open-circular form in the presence of 13 µM PPs. A pronounced lowering of the oxidatively induced OC form was observed. The OC form abundance was reduced back to below 1.5% in all samples containing PPs. The OC form was also reduced in the sample containing the native form of LDL. The OC form reduction was statistically significant between the samples treated with PPs or LDL versus the non-treated sample in the presence of H_2_O_2_ and Fe^2+^. Thus, it can be concluded, that both the LDL and the herein applied concentrations of quercetin and curcumin have an antioxidative action capable of reducing DNA radical-induced damage. The LDL-loaded PPs displayed the highest protective effect against oxidative DNA damage. Nevertheless, the differences in OC abundance were not significant between each of the PPs- compared to LDL-treated samples. The antioxidant potential of the native LDL in the isolated chemical system of the plasmid assay, versus the pro-oxidative character of LDL in cells, points to a different mode of action of LDL in these two instances. LDL acts as a chemical ROS scavenger in an isolated system, which is intuitive when we look at the LDL’s propensity to oxidize [43,44]. Furthermore, the PPs act as simple radical scavengers in a chemical system. At the same time, in the milieu of a living cell, PPs and LDL will display different effects as they interact with many other components present in the cell, and trigger various cellular signaling pathways. While cholesterol enhances ROS elevation in cells [45], a number of divergent curcumin effects have been shown, from protective as a free radical scavenger [46,47], to the pro-oxidative effect inducing apoptosis [48].

## 5. Conclusions

This research is a first, to our knowledge, attempt at preparatively loading LDL particles with polyphenols in order to estimate LDL availability as a PPs carrier. The LDL nanoparticle can be loaded with polyphenolic compounds. LDL capacity as a polyphenol carrier depends on the lipophilicity of the loaded PP. The PPs loading of the LDL disturbs the LDL receptor-dependent uptake in the cell, possibly due to the PPs interaction with the protein moieties, such as the lipophilic receptor-interacting protein region. Still, PPs-loaded LDL nanoparticles are non-specifically taken up in the cells. The level of this uptake allows LDL-entrapped Q and Cur to display a significant anti-ROS action, not observed for the pure polyphenols in the experimental conditions. 

## Figures and Tables

**Table 1:** The solubility and LogP values for Q, Cur, and CLA taken from DrugBank (www.drugbank.ca); logP values were calculated by the ALOGPS 2.1 freeware program (www.vcclab.org/lab/alogps).

Polyphenol	Water Solubility g/L	logP
Curcumin	0.00575	3.62
Quercetin	0.261	1.81
Chlorogenic acid	3.44	0.17

**Table 2:** The molar extinction coefficients (ε) for Q, Cur, and CLA, and the respective maximum absorption wavelengths, in methanol.

Polyphenol	Max (nm)	ε (mol^−1^cm^−1^)
Q	372	17864
Cur	422	52472
CLA	329	17971

Max—wavelength of the absorption maximum.

**Table 3:** The diameters (nm) and polydispersity indexes for the obtained LDL preparations.

Preparation	Average Diameter (nm)	Main Diameter (nm)	PDI
LDL	28.9	23.4	0.159
QLDL	29.7	23.0	0.195
CurLDL	29.6	24.5	0.185
CLALDL	30.9	22.8	0.195

**Table 4:** The PPs content in LDL after 2 and 11 h of incubation, per LDL volume. The protein content of the final PPLDL preparations was normalized to 3 mg/mL.

PP	Concentration (2 h, µM)	Concentration (11 h, µM)
Q	8.4	69.2
Cur	31.8	87.9
CLA	0	0

## Supplementary Materials

The following are available online at https://www.mdpi.com/1996-1944/13/22/5106/s1, Figure S1: DLS measurement of the size distribution of the obtained LDL preparation. Three reps of measurements are overlaid, Figure S2: DLS measurement of the size distribution of the obtained QLDL preparation (0.5 mg of Q per mL of LDL, 6 mg/mL of protein, incubated 11h at RT, with shaking in the dark). Three reps of measurements are overlaid, Figure S3: DLS measurement of the size distribution of the obtained CurLDL preparation (0.5 mg of Cur per mL of LDL, 6 mg/mL of protein, incubated 11 h at RT, with shaking in the dark). Three reps of measurements are overlaid, Figure S4: DLS measurement of the size distribution of the obtained CLALDL preparation (0.5 mg of CLA per mL of LDL, 6 mg/mL of protein, incubated 11 h at RT, with shaking in the dark). Three reps of measurements are overlaid.
