# Supplementary material for: New Polyphenol-Containing LDL Nano-Preparations in Oxidative Stress and DNA Damage: A Potential Route for Cell-Targeted PP Delivery
Source: Materials (Basel). 2020 Nov 12;13(22):5106. doi: 10.3390/ma13225106 (PMC7696759; doi:10.3390/ma13225106)
Supplement: Supplementary file 1 [file materials-13-05106-s001.pdf]

Supplementary Materials

New Polyphenol-Containing LDL Nano-Preparations in Oxidative Stress and DNA Damage: A Potential Route for Cell-Targeted PP Delivery

Hanna Lewandowska [1,*] and Monika Kalinowska [2]

[1] Centre for Radiation Research and Technology, Institute of Nuclear Chemistry and Technology, 16 Dorodna St., 03195 Warsaw, Poland

[2] Department of Chemistry, Biology and Biotechnology, Faculty of Civil Engineering and Environmental Sciences, Bialystok University of Technology, Wiejska 45E Street, 15351 Bialystok, Poland; m.kalinowska@pb.edu.pl

* Correspondence: h.lewandowska@ichtj.waw.pl; Tel.: +48-225-041-084

Received: 14 October 2020; Accepted: 10 November 2020; Published: date

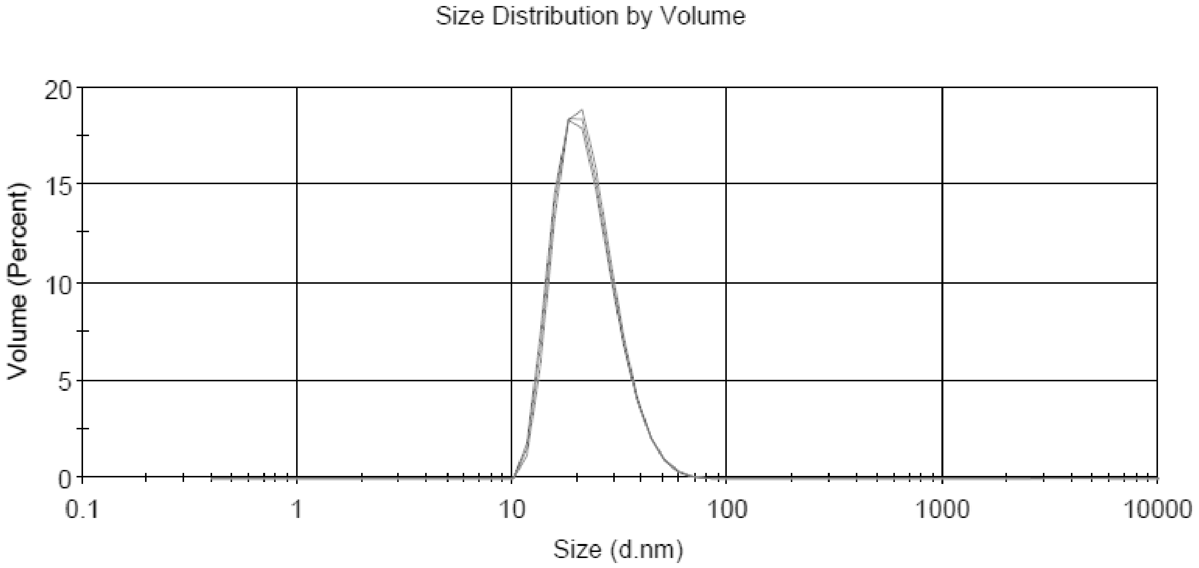

Figure S1. DLS measurement of the size distribution of the obtained LDL preparation. Three reps of measurements are overlaid.

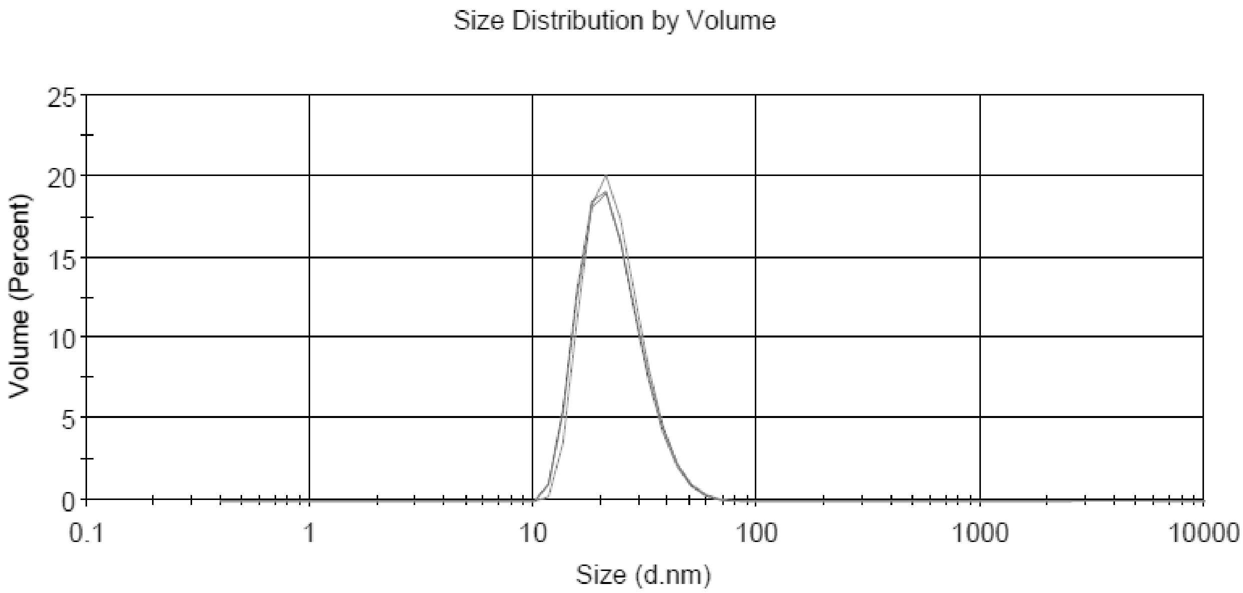

Figure S2. DLS measurement of the size distribution of the obtained QLDL preparation (0.5 mg of Q per mL of LDL, 6 mg/mL of protein, incubated 11h at RT, with shaking in the dark). Three reps of measurements are overlaid.

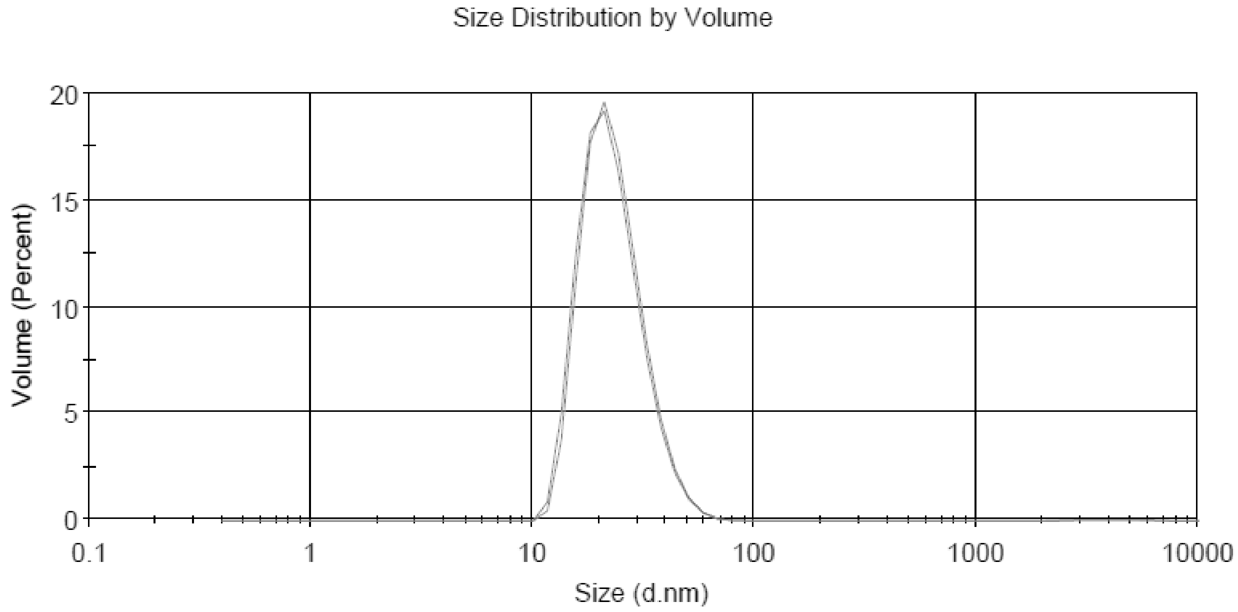

Figure S3. DLS measurement of the size distribution of the obtained CurLDL preparation (0.5 mg of Cur per mL of LDL, 6 mg/mL of protein, incubated 11h at RT, with shaking in the dark). Three reps of measurements are overlaid.

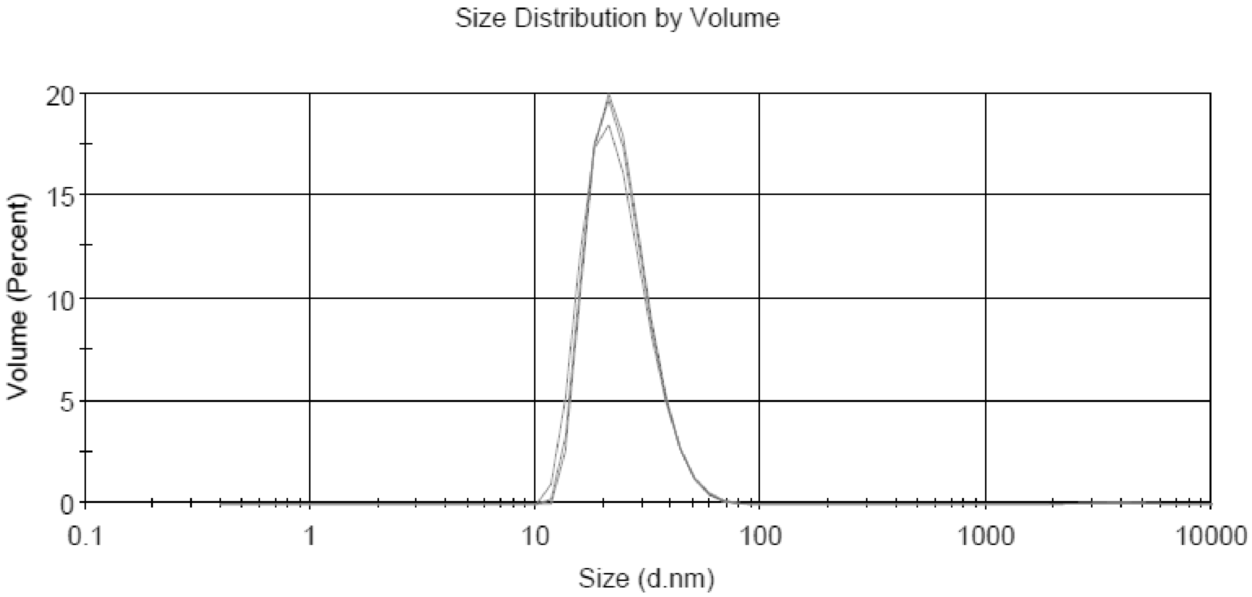

Figure S4. DLS measurement of the size distribution of the obtained CLALDL preparation (0.5 mg of CLA per mL of LDL, 6 mg/mL of protein, incubated 11h at RT, with shaking in the dark). Three reps of measurements are overlaid.

© 2020 by the authors. Submitted for possible open access publication under the terms and conditions of the Creative Commons Attribution (CC BY) license (http://creativecommons.org/licenses/by/4.0/).
